# Lepidotrichilins A and B, New Protolimonoids with Cytotoxic Activity from *Trichilia Lepidota* (Meliaceae)

**DOI:** 10.3390/molecules181012180

**Published:** 2013-09-30

**Authors:** Wagner da S. Terra, Ivo J. C. Vieira, Raimundo Braz-Filho, William R. de Freitas, Milton M. Kanashiro, Maria Conceição M. Torres

**Affiliations:** 1 Laboratório de Ciências Químicas, Universidade Estadual do Norte Fluminense Darcy Ribeiro, Avenida Alberto Lamego 2000, 28013-602, Campos dos Goytacazes, Rio de Janeiro, Brazil; 2 Laboratório de Biologia do Reconhecer, Universidade Estadual do Norte Fluminense Darcy Ribeiro, Avenida Alberto Lamego 2000, 28013-602, Campos dos Goytacazes, Rio de Janeiro, Brazil; 3 Departamento de Química Orgânica e Inorgânica, Universidade Federal do Ceará, 60021-970, Fortaleza, Ceará, Brazil

**Keywords:** *Trichilia lepidota*, Meliaceae, protolimonoids, spectral data, leukemic cell lines

## Abstract

Two novel protolimonoids, named lepidotrichilins A (**1**) and B (**2**), four known protolimonoids, 21,23-epoxy-7α-21α-dihydroxyapotirucalla-14,24-dien-3-one (**3**), 21,23-epoxy-7α-21β-dihydroxyapotiru-calla-14,24-dien-3-one (**4**), dysorone D (**5**), deoxy-flindissone (**6**), and the two steroids β-sitosterol (**7**) and stigmasterol (**8**) were identified in leaves of *Trichilia lepidota* subsp. *schumanniana* (Harms) T.D. Pennington. From wood the coumarin scopoletin (**9**) was isolated. The structures were established by NMR (1D ^1^H and ^13^C-NMR and 2D ^1^H-^1^H COSY, HMQC and HMBC), mass spectroscopy and infrared (IR) spectral data. The hexane and methanol extracts of the leaves, the protolimonoids lepidotrichilins A (**1**) and B (**2**) (IC_50_ 42.7 µg mL^−1^) and the protolimonoid deoxy-flindissone (**6**; IC_50_ 9.3 µgmL^−1^) exhibited significant cytotoxic activity against the MOLT-4 and U937 leukemic cell lines.

## 1. Introduction

*Trichilia lepidota* subsp. schumanniana (Harms) T.D. Pennington (known popularly as “Cedrinho” in Santa Catarina and Minas Gerais States, Brazil) is a tree of 3-6 meters in height. This wood of this species is used to make furniture [1]. The *Trichilia* genus consists of about 230 species distributed throughout tropical America and other countries ranging from Africa and Asia [2,3]. Previous investigations on the chemical constituents of the dichloromethane extract from the leaves of this species revealed the presence of hydrocarbons mixtures (C_29_H_60_, C_31_H_64_ and C_33_H_68_), sesquiterpenes, steroids, amino acids and other compounds derived from the terpene metabolic pathway [4].

Species of the genus *Trichilia* (Meliaceae) are known to contain varied limonoid structures (tetranortriterpenoids) and other terpenic metabolites which are responsible for various biological properties. Limonoids of various *Trichilia* with a wide spectrum of biological effects such as potential antiviral, analgesic, insecticidal, and insect growth inhibition activity have been documented [5,6]. Previous investigations involving limonoids isolated of another Meliaceae species, namely *Melia azedarach*, have demonstrated antiproliferative activity against an adenocarcinoma epithelial cell line A549 [7]. Additionally, it has been proved that limonoids from *Citrus* fruits demonstrated *in vitro* the capacity to induce apoptosis and to inhibit the proliferation of neuroblastoma cells [8].

**Figure 1:**
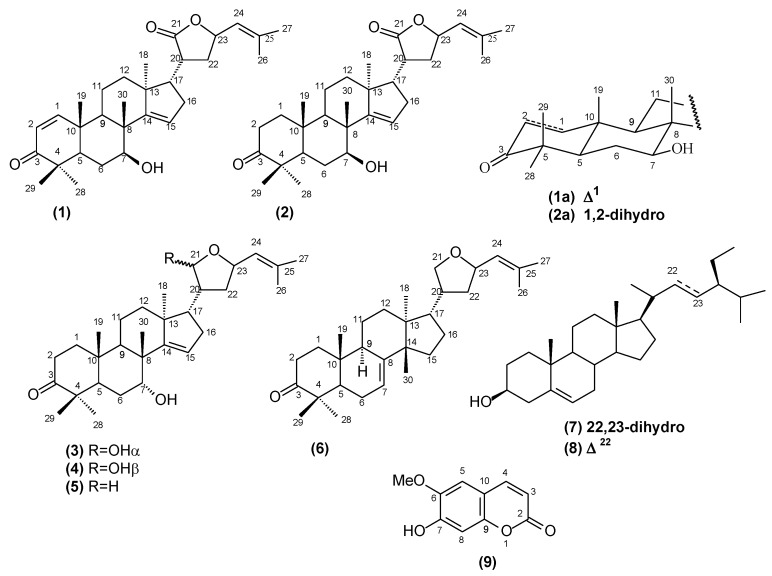
Chemical structures of the compounds isolated from *T. lepidota*.

In the present paper, we describe the isolation and characterization of the mixture of two new protolimonoids named lepidotrichilin A (**1**) and lepidotrichilin B (**2**), along with the known protolimonoids 21,23-epoxy-7α-21α-dihydroxyapotirucalla-14,24-dien-3-one (**3**), 21,23-epoxy-7α-21β-dihydroxyapotirucalla-14,24-dien-3-one (**4**), dysorane D (**5**) and deoxyflindissone (**6**), the steroids β-sitosterol (**7**) and stigmasterol (**8**) and the coumarin scopoletin (**9**), whose structures are summarized in Figure 1.

The structures of the known and new compounds were established on the basis of spectral data, mainly ^1^H- and ^13^C-NMR (1D and 2D), HRESIMS spectra and by comparison with literature data. This paper also describes the viability of human leukemic lineages cells U937 and MOLT-4 treated with the hexane and methanol extracts, the mixture of the protolimonoids lepidotrichilins A (**1**) and B (**2**) and the protolimonoid desoxyflindissone (**6**).

## 2. Results and Discussion

The hexane extract of *T. lepidota* leaves was eluted successively in several chromatographic columns affording a mixture of two new protolimonoids, lepidotrichilin A (**1**) and lepidotrichilin B (**2**), four known protolimonoids, 21,23-epoxy-7α-21α-dihydroxyapotirucalla-14,24-dien-3-one (**3**), 21,23-epoxy-7α-21β-dihydroxyapotirucalla-14,24-dien-3-one (**4**), dysorane D (**5**), deoxyflindissone (**6**) and the steroid β-sitosterol (**7**). These compounds were identified by comparison of their spectroscopic data (MS, IR, 1D- ^1^H and ^13^C-NMR, 2D-NMR) with those reported in the literature [9]. The methanol extract of *T. lepidota* leaves was processed on several chromatographic columns affording two steroids, β-sitosterol (**7**) and stigmasterol (**8**) and the protolimonoid deoxyflindissone (**6**). The methanol extract of *T. lepidota* wood affording the coumarin scopoletin (**9**).

The mixture of protolimonoids **1** and **2** was obtained as yellow oil. The IR spectrum (KBr disk) for the compounds showed bands at ν_max_ 3,438 cm^−1^, attributed to a hydroxyl group, 2,925 and 2,855, attributed to methyne and methylene groups, and 1,736 and 1,649 cm^−1^, attributed to two carbonyl groups at C-21(ester function) and C-3 (α,β-conjugated). The HRESIMS spectrum (positive mode) exhibited peaks at *m/z* 467.3200 ([M+H]^+^, calcd. *m/z* 467.3161) and 489.3021 ([M+Na]^+^, calcd. *m/z* 489.2981) corresponding to the molecular formula C_30_H_42_O_4_ of **1**, along with additional peaks at *m/z* 469.3363 ([M+H]^+^, calcd. *m/z* 469.3318) and 491.3188 ([M+Na]^+^, calcd. *m/z* 491.3137) corresponding to the molecular formula C_30_H_40_O_4_ of **2**, in accordance with ten (**1**) and nine (**2**) unsaturation degrees. Details of the molecular structures of compounds **1** and **2** were obtained by analysis of its ^1^H- and ^13^C-NMR spectra (Table 1) and from the observed ^1^H-^1^H-COSY, HMQC and HMBC correlations.

**Table 1:** ^13^C- (100 MHz) and ^1^H- (400 MHz) NMR data of the protolimonoids **1** and **2** in CDCl_3_, *δ* in ppm and multiplicities and *J* in Hz (in parenthesis), including results obtained by heteronuclear 2D shift-correlated HMQC (^1^*J*_HC_) and HMBC (^n^*J*_HC_ n = 2 and 3) *.

	1				2			
	HMQC		HMBC		HMQC		HMBC	
	*δ*_C_	*δ*_H_	^2^*J*_HC_	^3^*J*_HC_	*δ*_C_	*δ*_H_	^2^*J*_HC_	^3^*J*_HC_
**C**								
**3**	204.0	-		H-1; 3H-28; 3H-29	218.0	-		3H-28; 3H-29
**4**	44.2	-			46.7	-		
**8**	44.2	-			44.2	-		
**10**	41.8	-			37.2	-		
**13**	46.8	-			46.9	-		
**14**	161.0	-			161.0	-		
**21**	178.2	-			178.2	-		
**225**	140.0	-	3H-26; 3H-27		140.0	-	3H-26; 3H-27	
**CH**								
**1**	158.9	714 (*d*, 10.5)		3H-19	-	-		
**2**	125.4	5.83 (*d*, 10.5)			-	-		
**5**	44.6	2.40 (*m*)			46.7	2.40 (*m*)		
**7**	72.1	3.98 (*d*, 11.7)		3H-30	71.6	3.98 (*d*, 11.7)		3H-30
**9**	40.8			3H-19; 3H-30	41.1			3H-19;3H-30
**15**	119.6	5.51 (*m*)			119.6	5.51 (*m*)		
**17**	54.7	2.28 (*m*)		3H-18	54.0	2.28 (*m*)		3H-18
**20**	36.8	1.84 (*m*)			36.8	1.84 (*m*)		
**23**	75.3	5.19 (*brd*, 8.8)			75.3	5.19 (*brd*, 8.8)		
**24**	122.8	5.05 (*m*)		3H-26; 3H-27	122.8	5.05 (*m*)		
**CH_2_**								
**1**	-	-			38.5			3H-19
**2**	-	-			32.2	2.28		
**6**	24.8				24.3			
**11**	16.2				16.2			
**12**	32.0	2.32 (*m*)		3H-18	32.0	2.32 (*m*)		3H-18
**16**	33.9	2.10 (*m*)			33.9	2.10 (*m*)		
**22**	35.0				35.1			
**CH_3_**								
**18**	18.9	1.16 (*s*)			20.2	1.17 (*s*)		
**19**	24.9	1.02 (*s*)			14.9	1.02 (*s*)		
**26**	18.4	1.75 (*brs*)		3H-27	18.4	1.75 (*brs*)		3H-27
**27**	25.7	1.78 (*brs*)		3H-26	25.7	1.78 (*brs*)		3H-26
**28**	27.1	1.09 (*s*)			27.7	1.05 (*s*)		
**29**	26.1	1.15 (*s*)			27.2	1.05 (*s*)		
**30**	21.2	1.04 (*s*)			21.5	1.04 (*s*)		

^a^ *Number of hydrogens bound to carbon atoms deduced by comparative analysis of {^1^H}- and APT-^13^C NMR spectra. Chemical shifts and coupling constants (*J*) obtained of 1D ^1^H-NMR spectrum. Superimposed ^1^H signals are described without multiplicity and chemical shifts deduced by HMQC, HMBC and ^1^H-^1^H-COSY spectra.

Comparative analysis of the ^13^C-NMR spectra ({^1^H}- and APT) of the protolimonoids **1** and **2** was used to recognize the signals corresponding to eight non-hydrogenated [four sp^3^ and four sp^2^, including two carbonyl groups at *δ*_C_ 204.0 (α,β-unsaturated C-3 of **1**) and 218.0 (C-3 of **2**)], ten to **1** (four sp^2^ and six sp^3^ including two linked to oxygen atom) and eight to **2** (two sp^2^ and six sp^3^ including two linked to oxygen atom) methine, five to **1** (all sp^3^) and seven to **2** (all sp^3^) methylene and seven methyl (**1** and **2**) carbon atoms, corresponding to 30 carbon signals for each compound (Table 1): (C=O)(COO)(=C)_2_(C)_4_(=CH)_4_(CH-O)(CH-OH)(CH)_4_(CH_2_)_5_(CH_3_)_7_ = C_30_H_42_O_4_ for **1** [ten degrees of unsaturation: tetracyclic skeleton, the presence of two carbonyl groups at *δ*_C_ 204.0 (α,β-unsaturated ketone C-3) and *δ*_C_ 178.2 (lactone C-21) and three double bonds] and (C=O)(COO)(=C)_2_(C)_4_(=CH)_2_(CH-O)(CH-OH)(CH)_6_(CH_2_)_5_(CH_3_)_7_ = C_30_H_42_O_4_ for **2** [nine degrees of unsaturation: tetracyclic skeleton, the presence of two carbonyl groups at *δ*_C_ 218.0 (ketone C-3) and *δ*_C_ 178.2 (lactone C-21) and two double bonds]. Thus, seven methyl groups and eight non-hydrogenated carbon atoms (four sp^2^ and four sp^3^) on each compound **1** and **2**, revealed clearly the principal differences anticipated by the presence of a double bond between the carbon atoms 1 and 2 of **1** [*δ*_C_ 158.9/*δ*_H_ 7.14 (*J* = 10.5 Hz, CH-1) and *δ*_C_ 125.4/*δ*_H_ 5.83 (*J* = 10.5 Hz, CH-2)] and their corresponding absence in **2** suggested by two additional methylene signals [*δ*_C_ 38;5 (CH_2_-1) and 32.2 (CH_2_-2)]. The signals corresponding to methine carbons linked to an oxygen atom HOCH-7 were observed at *δ*_C_ 72.1/*δ*_H_ 3.98 (*d*, *J* = 11.7 Hz, **1**) and 71.6/*δ*_H_ 3.98 (*d*, *J* = 11.7 Hz. **2**), using a value of *J* = 11.7 Hz (axial-axial interaction) to locate the hydroxyl group at OH as equatorial (**1a** and **2a**) and HMQC spectrum to confirm these deductions by direct heteronuclear (^1^*J*_HC_) correlations. The signals (*δ*_C_/*δ*_H_) at *δ*_C_ 75.3/*δ*_H_ 5.19 (*brd*, *J* = 8.8 Hz) were attributed to CH-23 of **1** and **2**. Analogous results for **1** and **2** were attributed to carbon signals of the double bonds located between C-14 (*δ*_C_ 161.0) and CH-15 [*δ*_C_ 119.6/*δ*_H_ 5.51 (*m*)] and between CH-24 [*δ*_C_ 122.8/*δ*_H_ 5.05 (*m*)] and C-25 (*δ*_C_ 140.0). The proposed structures of **1** and **2** were confirmed by analyses of the 2D NMR spectra (HMBC, HMQC and ^1^H-^1^H-COSY), shown in Table 1.

The location of a hydroxyl group at CH-7 in both compounds was confirmed by HMBC correlations of the signals corresponding to CH-7 of **1** (*δ*_C_ 72.1/*δ*_H_ 1.04 (3H-30, ^3^*J*_HC_) and **2** (*δ*_C_ 71.6/*δ*_H_ 1.04 (3H-30, ^3^*J*_HC_). The value from the coupling constants (*J* = 11 7 Hz) corresponding to a *vicinal* interaction (^3^*J*_H,H_) between the hydrogens H-7 (*δ*_H_ 3.98, *d*, 11.7 Hz) with H-6ax is consistent with the axial-axial interaction shown in **1**/**1a** and **2**/**2a**. The significant differences between **1** and **2** can be attributed to the presence of the one more endocyclic (*J* = 10.5 Hz, *cis*) double bond in the ring-A (CH-1/CH-2) of **1**. The protolimonoids **1** and **2** were named lepidotrichilin A (**1**) and lepidotrichilin B (**2**, 1,2-dihydro-lepidotrichilin A).

The compounds **3**, **4** and **5** were obtained as colorless crystals, their ^13^C-NMR spectra showed 30 carbon signals. The compounds **3**, **4** and **5** were identified as 21,23-epoxy-7α-21α-dihydroxy-apotirucalla-14,24-dien-3-one, and 21,23-epoxy-7α-21β-dihydroxyapotiru-calla-14,24-dien-3-one previously isolated from *Luvunga sarmentosa* [10] and dysorane D isolated from *Dysoxylum roseum* [11].

The protolimonoid **6** was obtained as purple crystals. The molecular weight was deduced from a molecular ion peak at *m/z* 438.3498 allowing the assignment of the molecular formula C_30_H_46_O_2_. The IR spectrum of compound **6** showed absorptions at 1,732 cm^−1^, attributed to a carbonyl group (C-3), 800, 959, 1053 and 1,377 cm^−1^, attributed to a stretch of the C-O bonds in the ether function. It revealed the presence of one carbonyl functionality at *δ*_C_ 217.1 (C-3), seven methyl groups, nine methylene groups (CH_2_), seven methine groups (CH) including a carbon linked to an oxygen atom at (CH-23) *δ*_C_ 74.7/*δ*_H_ 4.61 (*dt*, *J* = 8.5 and 6.3 Hz) and seven nonhydrogenated carbons (C): four sp^3^ and three sp^2^. This compound have two double bonds between *δ*_C_ 118.3 (CH-7) and *δ*_C_ 145.7 (C-8); *δ*_C_ 127.0 (CH-24) and *δ*_C_ 135.3 (C-25). The protolimonoid **6** was identified as deoxyflindissone previously isolated from *Cornus walteri* [12] and synthesized by reduction of flindissol with lithium aluminum hydride following the oxidation of the deoxyflindissol [13]. Compounds **7**, **8** and **9** were were identified as β-sitosterol (**7**), stigmasterol (**8**) [14] and scopoletin (**9**) by comparison of their ^1^H- and ^13^C-NMR and GC/MS spectra with data from the literature [15].

Cellular viability after treatment with purified compounds and plant extracts was evaluated by MTT metabolization. Reduction in cell viability in both cell lineage submitted to treatment to all extract and isolated compounds were shown on Figure S16 (see the Supplementary Material) and summarized in Table 2. In all cell viability experiments DMSO was used as a normal cell culture control and meaningless cell death was observed, therefore those controls treatments were depicted as 100% of cell viability compared to the cell culture treated with compounds and extracts. Concerning cell death control 15 µgmL^−1^ of cisplatin was used to treat the cells and 90% of cell death was observed. Based on both leukemia cell viability analyses, our data suggest that protolimonoid **6** would be more active than the protolimonoid compounds **1** and **2**.

Our results are in agreement with the activity limonoids isolated from *Citrus* [8], which were able to reduce viability of neuroblastoma cell line SH-SY5Y. In contrast to our data, the limonoids isolated from *Toona ciliata* var. *pubescens* [16] were inactive or had weak activity on cancer and non-cancer cell line, but were able to inhibit CDC25B dual specificity phosphatase. Altogether, these data suggest a range of biological activity associated with a variety of limonoids molecules structures. Table 2 lists the IC_50_ values for cellular viability and LDH release. Analysis of the IC_50_ values shows that the isolated protolimonoid **6** was more efficient at inducing cellular cytotoxicity than the new protolimonoids **1** and **2**.

**Table 2:** IC_50_ determination for cellular viability and LDH release.

	U937-IC_50_ (µgmL^−1^)		MOLT4-IC_50_ (µgmL^−1^)	
Extract/Compound	Viability-MTT	LDH-Release	Viability-MTT	LDH-Release
**1** and **2**	48.0	681.4	42.7	315.0
**6**	≤9.3	32.8	≤9.3	19.8
Methanol extract	61.4	528.3	47.4	472.7
Hexane extract	<62.5	<62.5	<62.5	88.2

The lower IC_50_ value 9.3 µgmL^−1^ was observed for protolimonoid **6** for both cells line U937 and MOLT-4. Among the *T. lepidota* extracts the hexane ones were more efficient than the methanol ones. Comparing the values of IC_50_ for LDH release and cellular cytotoxicity we can observe that the sample concentration to induce LDH release was much higher than the sample concentration to induce cytotoxicity. Those data suggested that cell death occurred by apoptosis. Other studies on limonoids showed that these compounds were able to reduce cell viability and induce apoptosis in some cancer cells. Tian *et al*. showed that four isolated limonoids from *Citrus reticulata* induced cell death by apoptosis in breast cancer cell line (MCF-7) [17]. Additionally neuroblastoma cell culture was shown to die by apoptosis when incubated with limonoids isolated from *Citrus* [8].

## 3. Experimental

### 3.1. General Procedures

The IR spectrum was measured on a Shimadzu IR affinity spectrometer. ^1^H- and ^13^C-NMR, APT-NMR and 2D-NMR spectra (^1^H-^1^H-COSY, HMQC and HMBC) were recorded on a Jeol Eclipse-400 (400 MHz for ^1^H and 100 MHz for ^13^C), using CDCl_3_ as the solvent. High resolution electrospray ionization mass spectra (HRESIMS) were acquired using a LCMS-IT-TOF (Shimadzu) spectrometer, at the Departmento de Química Orgânica e Inorgânica, UFC, Fortaleza, Ceará, Brazil.

### 3.2. Plant Material

The leaves (1.9 kg) and wood (2.4 kg) of *T. lepidota* subsp. schumanniana (Harms) T. D. Pennington were collected in November 2006 at Vale Doce Forest Reserve, Linhares City, Espírito Santo State, Brazil. A voucher specimen (CVRD 8743) is deposited at Vale Doce Forest Reserve herbarium, Linhares, Espírito Santo State, Brazil.

### 3.3. Extraction and Isolation

The leaves (1.9 Kg) were dried, powdered and extracted consecutively with hexane (3 L) and methanol (3 L) for three time and ambient temperature. The hexane extract of *T. lepidota* leaves (24.8 g) was subjected to chromatography on silica gel 60G (63-200 µm, Merck), using a gradient of hexane-ethyl acetate. Initially 100% hexane was used, and then the composition was changed to 100% ethyl acetate in 5% increments, affording 13 fractions. The fraction 3 (1.32 g) was rechomatographed (hexane-ethyl acetate) affording 10 fractions. The fraction 3.4 (99.0 mg) was subjected to preparative chromatography, eluted with an ethyl acetate-hexane mixture (35:75, *v/v*), affording protolimonoid **6** (14.8 mg). Fraction 4 (3.17 g) after column chromatography using a gradient of hexane-ethyl acetate (initially 90% hexane was used, and then the composition was changed to 100% ethyl acetate in 5% increments), affording a mixture of steroids **7** and **8** (923.0 mg). Fraction 8 (1.70 g) after column chromatography using a gradient of hexane-ethyl acetate (initially 90% hexane was used, and then the composition was changed to 100% ethyl acetate in 5% increments), affording 11 fractions. The crystals of fraction 8.6 were collected affording protolimonoids **3**, **4** and **5** (79.3 mg). Fraction 10 (1.09 g) after column chromatography using a gradient of CH_2_Cl_2_-MeOH (initially 90% CH_2_Cl_2_ was used, and then the composition was changed to 100% dichloromethane in 1% increments, and then CH_2_Cl_2_-MeOH (9:1, *v/v*)) affording six fractions. The fraction 10.2 (97.8 mg) after column chromatography using a gradient of hexane-ethyl acetate (initially 90% hexane was used, and then the composition was changed to 100% ethyl acetate in 5% increments) affording a mixture a new protolimonoids **1** (37.2%) and **2** (62.8%; 14.3 mg in total).

The methanol extract of *T. lepidota* leaves (17.35 g) was fractionated on silica gel 60G (63-200 µm, Merck), using a gradient of CH_2_Cl_2_-MeOH. Initially 100% dichloromethane was used, and then the composition was changed to 5% methanol in 1% increments affording eight fractions. Fraction 8.4 affording a mixture of steroids **7** and **8** (392.7 mg). Fraction 8.5 (609.2 mg) was subjected to chromatography on silica gel 60G (63-200 µm, Merck), using a gradient of CH_2_Cl_2_-MeOH, mixed with activated charcoal. Initially 100% dichloromethane was used, and then the composition was changed to 5% methanol in 1% increments affording the protolimonoid desoxyflindissone **6** (27.7 mg).

The wood (1.5 kg) was dried, powdered and extracted with methanol (3 L) for three time and ambient temperature. The extract of *T. lepidota* wood (29.47 g) was first submitted to a liquid-liquid partition with H_2_O-CH_2_Cl_2_ (1:3). Part of the dichloromethane fraction (150.0 mg) was fractionated by column chromatography using a gradient of CH_2_Cl_2_-MeOH. Initially 100% dichloromethane was used, and then the composition was changed to 5% methanol in 1% increments, affording coumarin **9** (17.4 mg).

### 3.4. Spectroscopic Data

*Lepidotrichilin A* (**1**)* + Lepidotrichilin B* (**2**). Obtained as yellow oil. HRESIMS (positive mode) *m/z* 467.3200 ([M+H]^+^, calcd. *m/z* 467.3161) and 489.3021 ([M+Na]^+^, calcd. *m/z* 489.2981) **1**); *m/z* 469.3363 ([M+H]^+^, calcd. *m/z* 469.3318) and 491.3188 ([M+Na]^+^, calcd. *m/z* 491.3137) **2**); ^1^H- and ^13^C-NMR data, *see* Table 1.

*21,23-Epoxy-7α-21α-dihydroxyapotirucalla-14,24-dien-3-one* (**3**): Obtained as colorless crystals; ^13^C-NMR *δ* (ppm): 217.4 (C-3); 46.9 (C-4); 44.0 (C-8); 37.3 (C-10); 46.8 (C-13); 161.9 (C-14); 135.2 (C-25); 46.5 (CH-5); 72.2 (CH-7); 40.9 (CH-9); 119.9 (CH-15); 58.8 (CH-17); 48.2 (CH-20); 90.3 (CH-21); 74.7 (CH-23); 126.6 (CH-24); 38.4 (CH_2_-1); 33.6 (CH_2_-2); 24.9 (CH_2_-6); 16.4 (CH_2_-11); 33.7 (CH_2_-12); 35.5 (CH_2_-16); 38.2 (CH_2_-22); 19.5 (CH_3_-18); 14.9 (CH_3_-19); 18.2 (CH_3_-26); 25.9 (CH_3_-27); 26.4 (CH_3_-28); 21.3 (CH_3_-29); 27.3 (CH_3_-30). ^1^H-NMR *δ* (ppm): (2.10; *m*; H-5); (3.96; *s*; H-7); (2.00; *m*; H-9); (5.49; *s*; H-15); (1.65; *m*; H-17); (2.38; *m*; H-20); (5.06; *m*; H-21); (4.62; *m*; H-23); (5.21; *d*; *J* = 8.8 Hz; H-24); (1.48 and 1.76; *m*; 2H-1); (2.38 and 2.50; *m*; 2H-2); (1.80 and 1.32; *m*; 2H-6); (1.60 and 1.54; *m*; 2H-11); (1.68 and 1.40; *m*; 2H-12); (2.18; *m*; 2H-16); (1.70; *m*; 2H-22); (1.02; *s*; 3H-18); (1.00; *s*; 3H-19); (1.69; *s*; 3H-26); (1.72; *s*; 3H-27); (1.10; *s*; 3H-28); (1.05; *s*; 3H-29); (1.09; *s*; 3H-30).

*21,23-Epoxy-7α-21β-dihydroxyapotirucalla-14,24-dien-3-one* (**4**): Obtained as colorless crystals; ^13^C-NMR *δ* (ppm): 217.4 (C-3); 46.9 (C-4); 44.0 (C-8); 37.3 (C-10); 46.8 (C-13); 161.9 (C-14); 135.2 (C-25); 46.5 (CH-5); 72.2 (CH-7); 40.9 (CH-9); 119.9 (CH-15); 58.8 (CH-17); 48.2 (CH-20); 91.6 (CH-21); 74.7 (CH-23); 126.6 (CH-24); 38.4 (CH_2_-1); 33.6 (CH_2_-2); 24.9 (CH_2_-6); 16.4 (CH_2_-11); 33.7 (CH_2_-12); 35.5 (CH_2_-16); 38.2 (CH_2_-22); 19.5 (CH_3_-18); 14.9 (CH_3_-19); 18.2 (CH_3_-26); 25.9 (CH_3_-27); 26.4 (CH_3_-28); 21.3 (CH_3_-29); 27.3 (CH_3_-30). ^1^H-NMR *δ* (ppm): (2.10; *m*; H-5); (3.96; *s*; H-7); (2.00; *m*; H-9); (5.49; *s*; H-15); (1.65; *m*; H-17); (2.38; *m*; H-20); (5.06; *m*; H-21); (4.62; *m*; H-23); (5.21; *d*; *J* = 8.8 Hz; H-24); (1.48 and 1.76; *m*; 2H-1); (2.38 and 2.50; *m*; 2H-2); (1.80 and 1.32; *m*; 2H-6); (1.60 and 1.54; *m*; 2H-11); (1.68 and 1.40; *m*; 2H-12); (2.18; *m*; 2H-16); (1.70; *m*; 2H-22); (1.02; *s*; 3H-18); (1.00; *s*; 3H-19); (1.69; *s*; 3H-26); (1.72; *s*; 3H-27); (1.10; *s*; 3H-28); (1.05; *s*; 3H-29); (1.09; *s*; 3H-30).

*Dysorone* *D* (**5**): Obtained as colorless crystals; ^13^C-NMR *δ* (ppm): 217.4 (C-3); 46.9 (C-4); 44.0 (C-8); 37.3 (C-10); 46.8 (C-13); 161.9 (C-14); 135.2 (C-25); 46.5 (CH-5); 72.2 (CH-7); 40.9 (CH-9); 119.9 (CH-15); 58.8 (CH-17); 40.3 (CH-20); 74.7 (CH-23); 126.6 (CH-24); 38.4 (CH_2_-1); 33.6 (CH_2_-2); 24.9 (CH_2_-6); 16.4 (CH_2_-11); 33.7 (CH_2_-12); 35.5 (CH_2_-16); 72.3 (CH_2_-21); 38.2 (CH_2_-22); 19.5 (CH_3_-18); 14.9 (CH_3_-19); 18.2 (CH_3_-26); 25.9 (CH_3_-27); 26.4 (CH_3_-28); 21.3 (CH_3_-29); 27.3 (CH_3_-30). ^1^H-NMR *δ* (ppm): (2.10; *m*; H-5); (3.96; *s*; H-7); (2.00; *m*; H-9); (5.49; *s*; H-15); (1.65; *m*; H-17); (2.38; *m*; H-20); (4.62; *m*; H-23); (5.21; *d*; *J* = 8.8 Hz; H-24); (1.48 and 1.76; *m*; 2H-1); (2.38 and 2.50; *m*; 2H-2); (1.80 and 1.32; *m*; 2H-6); (1.60 and 1.54; *m*; 2H-11); (1.68 and 1.40; *m*; 2H-12); (2.18; *m*; 2H-16); [(4.08; *t*, *J* = 7.0 Hz; H-21a); (3.25; *td*; *J* = 7.0 and 8.2 Hz; H-21b)]; (1.70; *m*; 2H-22); (1.02; *s*; 3H-18); (1.00; *s*; 3H-19); (1.69; *s*; 3H-26); (1.72; *s*; 3H-27); (1.10; *s*; 3H-28); (1.05; *s*; 3H-29); (1.09; *s*; 3H-30).

*Deoxyflindissone* (**6**): Purple crystals; m.p. 123 ºC; ^13^C-NMR *δ* (ppm): 217.1 (C-3); 48.1 (C-4); 145.7 (C-8); 35.3 (C-10); 43.9 (C-13); 50.9 (C-14); 135.3 (C-25); 51.1 (CH-5); 118.3 (CH-7); 48.5 (CH-9); 52.6 (CH-17); 42.4 (CH-20); 74.7 (CH-23); 127.0 (CH-24); 38.9 (CH_2_-1); 35.3 (CH_2_-2); 24.5 (CH_2_-6); 17.9 (CH_2_-11); 34.5 (CH_2_-12); 32.1 (CH_2_-15); 28.0 (CH_2_-16); 72.3 (CH_2_-21); 38.9 (CH_2_-22); 12.9 (CH_3_-18); 22.8 (CH_3_-19); 18.3 (CH_3_-26); 26.0 (CH_3_-27); 24.8 (CH_3_-28); 21.8 (CH_3_-29); 27.5 (CH_3_-30). ^1^H-NMR *δ* (ppm): (1.68; *m*; H-5); (5.31; *d*; *J* = 3.0 Hz; H-7); (2.32; *m*; H-9); (1.75; *m*; H-17); (2.25; *m*; H-20); (4.61; *td*; *J* = 8.5 and 6.3 Hz; H-23); (5.21; *d*; *J* = 8.5 Hz; H-24); (1.50 and 2.01; *m*; 2H-1); [(2.75; *ddd*; *J* = 14.1; 9.0 and 5.5 Hz; H-2a); (2.25; *m*; H-2b)]; (2.30 and 2.10; *m*; 2H-6); (1.80 and 1.60; *m*; 2H-11); (1.65 and 1.60; *m*; 2H-12); (1.70; *m*; 2H-15); (1.90 and 1.40; *m*; 2H-16); [(4.00; *dd*, *J* = 7.0 and 9.0 Hz; H-21a); (3.24; *t*; *J* = 9.0 Hz; H-21b)]; (1.78 and 1.68; *m*; 2H-22); (1.02; *s*; 3H-18); (1.12; *s*; 3H-19); (1.75; *brs*; 3H-26); (1.69; *brs*; 3H-27); (1.05; *s*; 3H-28); (0.83; *s*; 3H-29); (1.01; *s*; 3H-30). HR-ESIMS (positive mode) (*m/z*) 439.3402 [M+H]+ (calcd. 439.3576); 421.3313 [M−H_2_O+H]+ ((calcd. 4213470); 313.2400 [M−H_2_O−C_8_H_12_+H]+ (calcd. 313.2531); 295.2288 [M−2H_2_O−C_8_H_12_+H]+ (calcd. 295.2425); 403.3233 [M−2H_2_O+H]+ (calcd. 403.3364).

*β-Sitosterol* (**7**): ^13^C-NMR *δ* (ppm): 140.8 (C-5); 36.5 (C-10); 42.4 (C-13); 71.8 (CH-3); 121.7 (CH-6); 31.9 (CH-8); 50.2 (CH-9); 56.8 (CH-14); 56.1 (CH-17); 39.8 (CH-20); 45.9 (CH-24); 29.7 (CH-25); 37.3 (CH_2_-1); 31.9 (CH_2_-2); 42.4 (CH_2_-4); 34.0 (CH_2_-7); 21.1 (CH_2_-11); 39.8 (CH_2_-12); 24.3 (CH_2_-15); 28.9 (CH_2_-16); 31.9 (CH_2_-22); 26.2 (CH_2_-23); 23.1 (CH_2_-28); 12.0 (CH_3_-18); 19.4 (CH_3_-19); 18.8 (CH_3_-21); 19.8 (CH_3_-26); 19.8 (CH_3_-27); 11.9 (CH_3_-29). LRMS *m/z* (rel. int.): 414 (100%); 399; 381; 329; 273; 255; 231; 213.

*Stigmasterol* (**8**): ^13^C-NMR *δ* (ppm): 140.8 (C-5); 36.2 (C-10); 42.4 (C-13); 71.8 (CH-3); 121.7 (CH-6); 31.9 (CH-8); 50.2 (CH-9); 56.8 (CH-14); 56.1 (CH-17); 39.8 (CH-20); 138.3 (CH-22); 129.3 (CH-23); 50.2 (CH-24); 31.7 (CH-25); 37.3 (CH_2_-1); 31.9 (CH_2_-2); 42.4 (CH_2_-4); 34.0 (CH_2_-7); 21.1 (CH_2_-11); 39.8 (CH_2_-12); 24.3 (CH_2_-15); 28.9 (CH_2_-16); 25.4 (CH_2_-28); 12.0 (CH_3_-18); 19.4 (CH_3_-19); 19.1 (CH_3_-21); 21.2 (CH_3_-26); 19.8 (CH_3_-27); 12.0 (CH_3_-29). LRMS *m/z* (rel. int.): 412 (100%); 394; 273; 255; 231; 213.

*Scopoletin* (**9**): white amorphous powder; m.p. 206-207 ºC; ^13^C-NMR *δ* (ppm): 161.4 (C-2); 144.0 (C-6); 149.7 (C-7); 150.0 (C-9); 111.5 (C-10); 113.4 (CH-3); 143.3 (CH-4); 107.5 (CH-5); 103.2 (CH-8); 56.4 (OCH_3_). ^1^H-NMR *δ* (ppm): (6.27; *d*; *J* = 9.4 Hz; H-3); (7.60; *d*; *J* = 9.4 Hz; H-4); (6.85; *s*; H-5); (6.92; *s*; H-8); (3.95; *s*; OCH_3_). LRMS *m/z* (rel. int.): 192 (100%); 177 (63%); 164 (30%); 149 (59%); 121 (30%).

### 3.5. Cell Lines and Culture Conditions

Human Leukemia cells lineages U937 and MOLT-4 were grown in DMEM-12 (Gibco—BRL, Campos dos Goytacazes City, Rio de Janeiro State, Brazil) supplemented with 10% of bovine fetal serum and 20 μgmL^−1^ of gentamicin. The cultures were split every two or three days in 25 cm^2^ culture flask and maintained in incubator at 37 °C with 5% of CO_2_ and controlled humidity.

### 3.6. Extracts, Isolated Compounds and Treatments

The protolimonoids lepidotrichilin A (**1**) and B (**2**) were dissolved in DMSO and 500.0 μg·mL^−1^ was the highest concentration used for cell culture treatments. Due to the low solubility of the isolated protolimonoid desoxyflindissone (**6**) and the viscosity of the methanol and hexane extracts, 300 μL of DMSO was added to the each sample, which was homogenized and left standing for 24 h at room temperature. Samples were centrifuged at 11,000 × g for 10 min., then 200 µL of supernatant were collected and submitted to lyophilization. Freeze-dried samples were weighed and diluted in DMSO again. The starting dilution of protolimonoid desoxyflindissone (**6**) was 150 μg·mL^−1^, methanol extract 512.5 μg·mL^−1^ and hexane extract 1000.0 μg·mL^−1^. Cell viability assay was performed in 96 well plates with 5.0 × 10^5^ cellmL^−1^. As controls DMSO and cisplatin were included in all experiments.

### 3.7. Lactate Dehydrogenase Release Assay

Quantification of the enzyme lactate dehydrogenase (LDH) was performed in cell culture supernatants after treatment with the isolated compounds and plant extracts after the period of 48 hours of incubation at 37 °C. The enzyme LDH release was measured by the colorimetric assay from Doles (DLH Doles, Campos dos Goytacazes City, Brazil) according to the manufacturer's recommendation. For negative control, cells were treated with DMSO and positive control Triton was added to the culture. Optical density of the samples was measured in multichannel spectrophotometer with wavelength adjusted for 492 nm.

### 3.8. MTT Assay

Cell viability was determined by a 3-(4,5-dimethylthiazol-2-yl)-2,5-diphenyltetrazolium bromide (MTT) assay. MTT (5.0 mg·mL^−1^, 20 μL·well^−1^) was added to the cell culture after 48 h of treatments with different samples and incubated for 4 h at 37 °C. After incubation, 150 μL of medium from each one well was discarded and the purple crystals of MTT were dissolved by adding 100 μL of isopropanol solution with 0.0014% of HCl. After vigorous agitation the optical density of the samples was measured in multichannel spectrophotometer with wavelength adjusted for 570 nm. For negative and positive control cells were treated with DMSO and Triton, respectively.

### 3.9. Statistical Analyses

Data analysis was performed with GraphPad Prism Version 5.0 software with ANOVA and the powder-test of Tukey. The IC_50_ was calculated through the Non-linear regression test.

## 4. Conclusions

The hexane extract from the fruits of *T. lepidota* provided six protolimonoids **1**–**6**, two steroids **7**–**8** and one coumarin **9** which were isolated in a previous phytochemical investigation. The protolimonoids named lepidotrichilin A (**1**) and B (**2**) are described for the first time in the literature. The hexane and methanol extracts of the leaves, the protolimonoids lepidotrichilins A (**1**) and B (**2**) (IC_50_ 42.7 µg·mL^−1^) and the protolimonoid deoxyflindissone (**6**; IC_50_ 9.3 µg·mL^−1^) exhibited significant cytotoxic activity against the MOLT-4 and U937 leukemic cell lines.

## Supplementary Materials

Supplementary materials can be accessed at: http://www.mdpi.com/1420-3049/18/10/12180/s1.
