# Supplementary material for: Lepidotrichilins A and B, New Protolimonoids with Cytotoxic Activity from Trichilia Lepidota (Meliaceae)
Source: Molecules. 2013 Sep 30;18(10):12180–91. doi: 10.3390/molecules181012180 (PMC6270208; doi:10.3390/molecules181012180)

Supplementary Materials

Figure S1. IR spectrum of protolimonoids **1** and **2**.

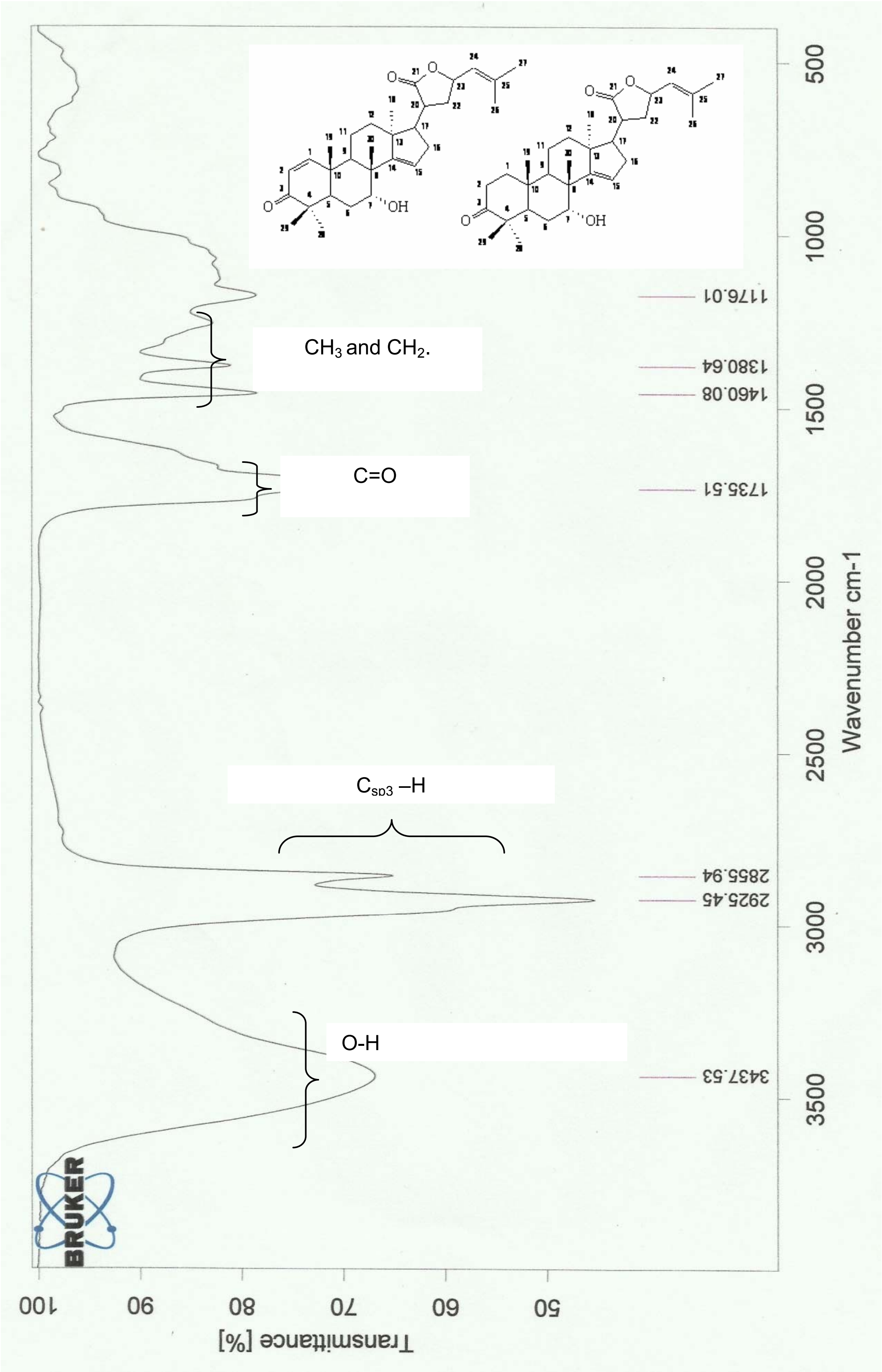

Figure S2. ^{1}H NMR spectrum of protolimonoids **1** and **2** (400 MHz, $CDCl_3$).

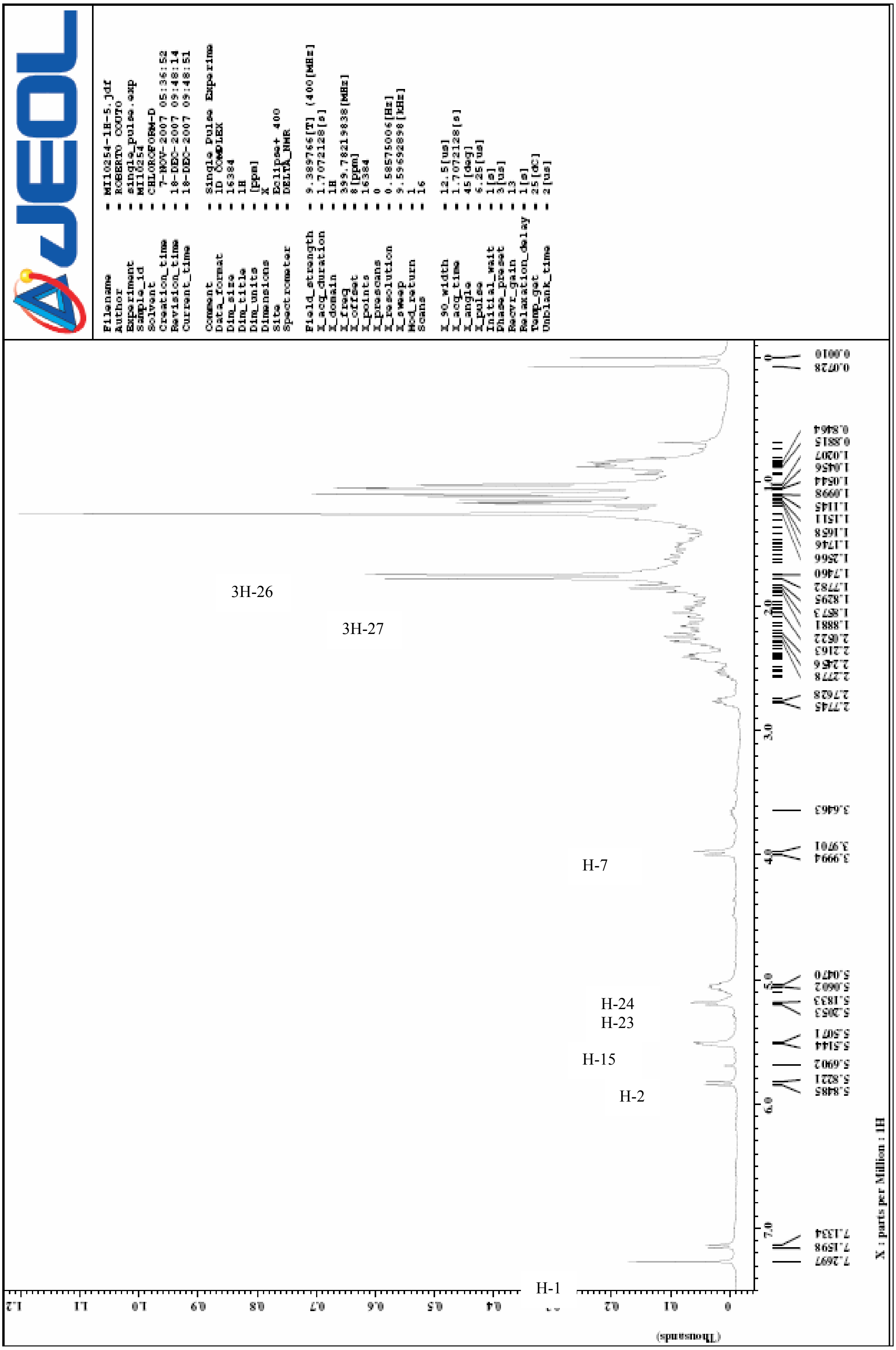

Figure S3. ^{1}H NMR spectrum of protolimonoids **1** and **2** (400 MHz, $CDCl_3$).

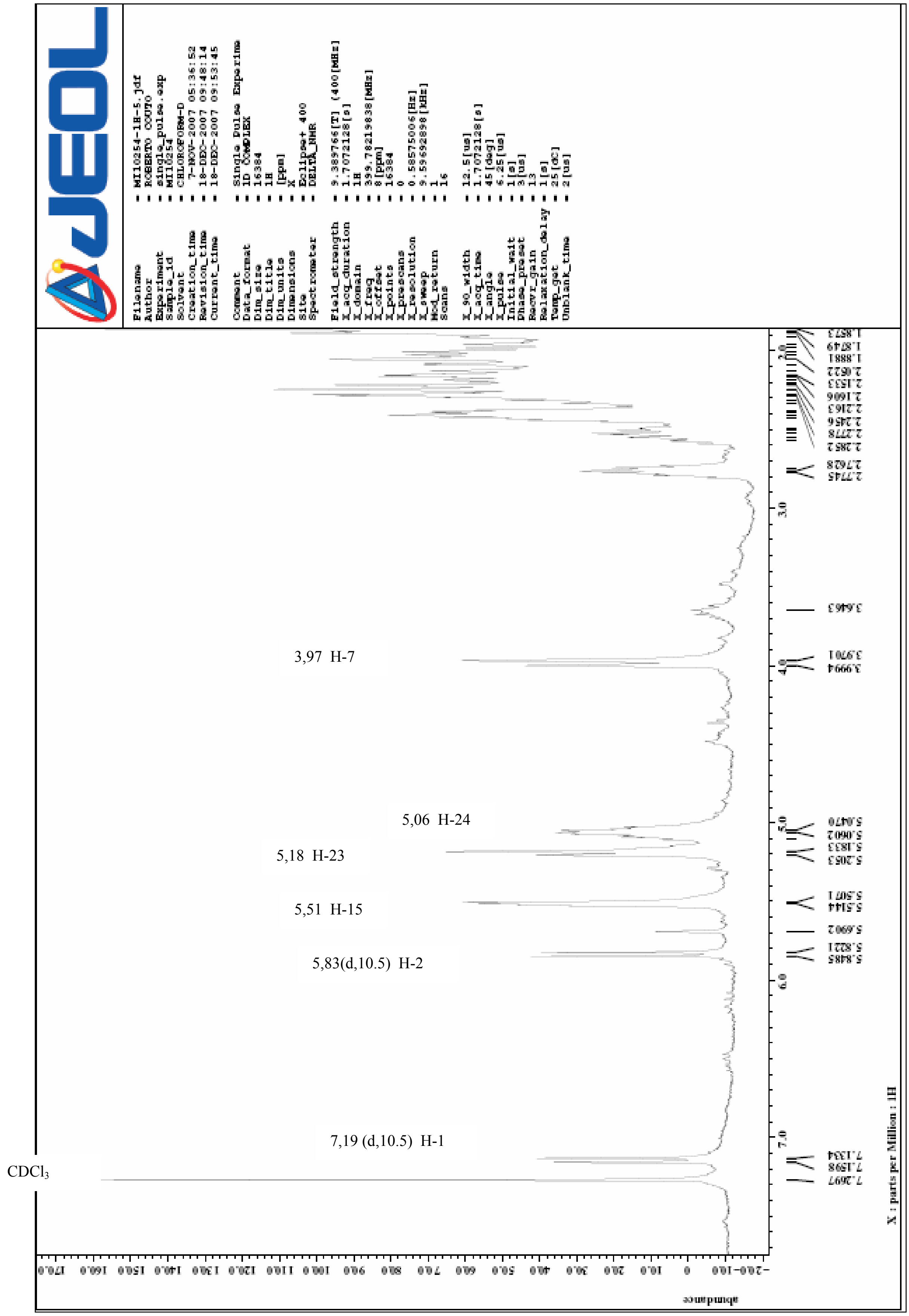

Figure S4. ^{13}C NMR-APT spectrum of protolimonoids **1** and **2** (100 MHz, $CDCl_3$).

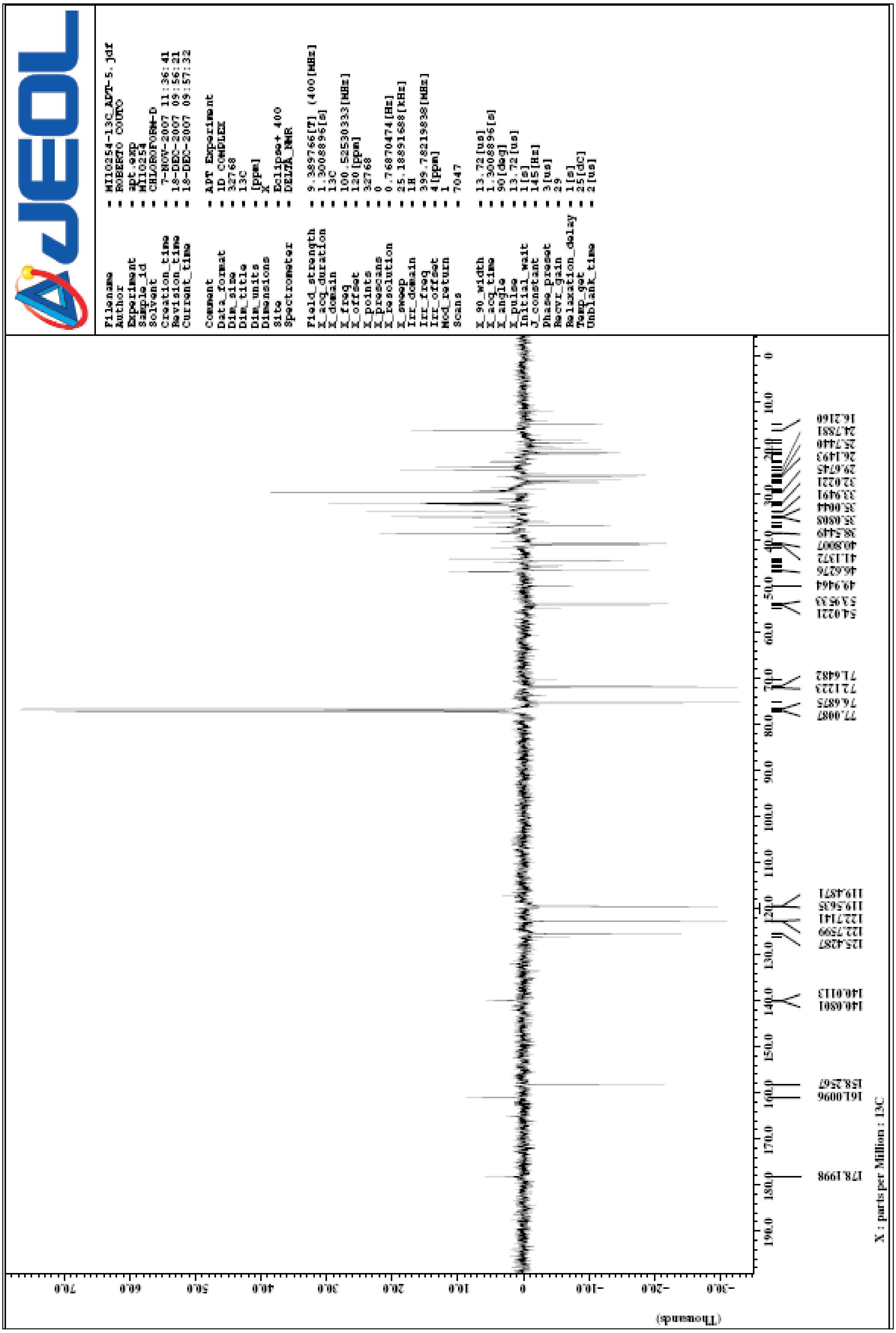

Figure S5. ^{13}C NMR-APT spectrum of protolimonoids **1** and **2** (100 MHz, $CDCl_3$).

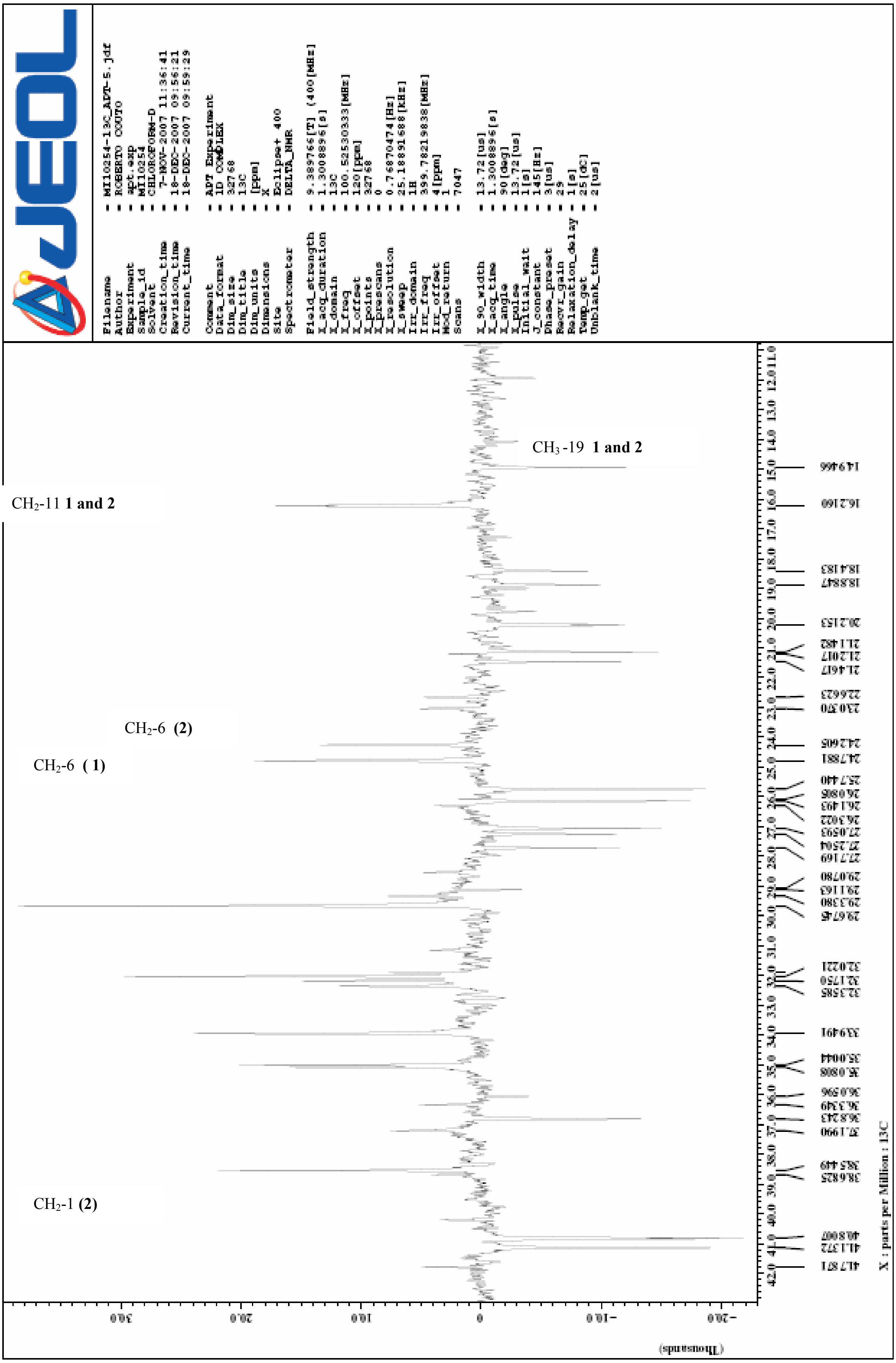

Figure S6. ^{13}C NMR-APT spectrum of protolimonoids **1** and **2** (100 MHz, $CDCl_3$).

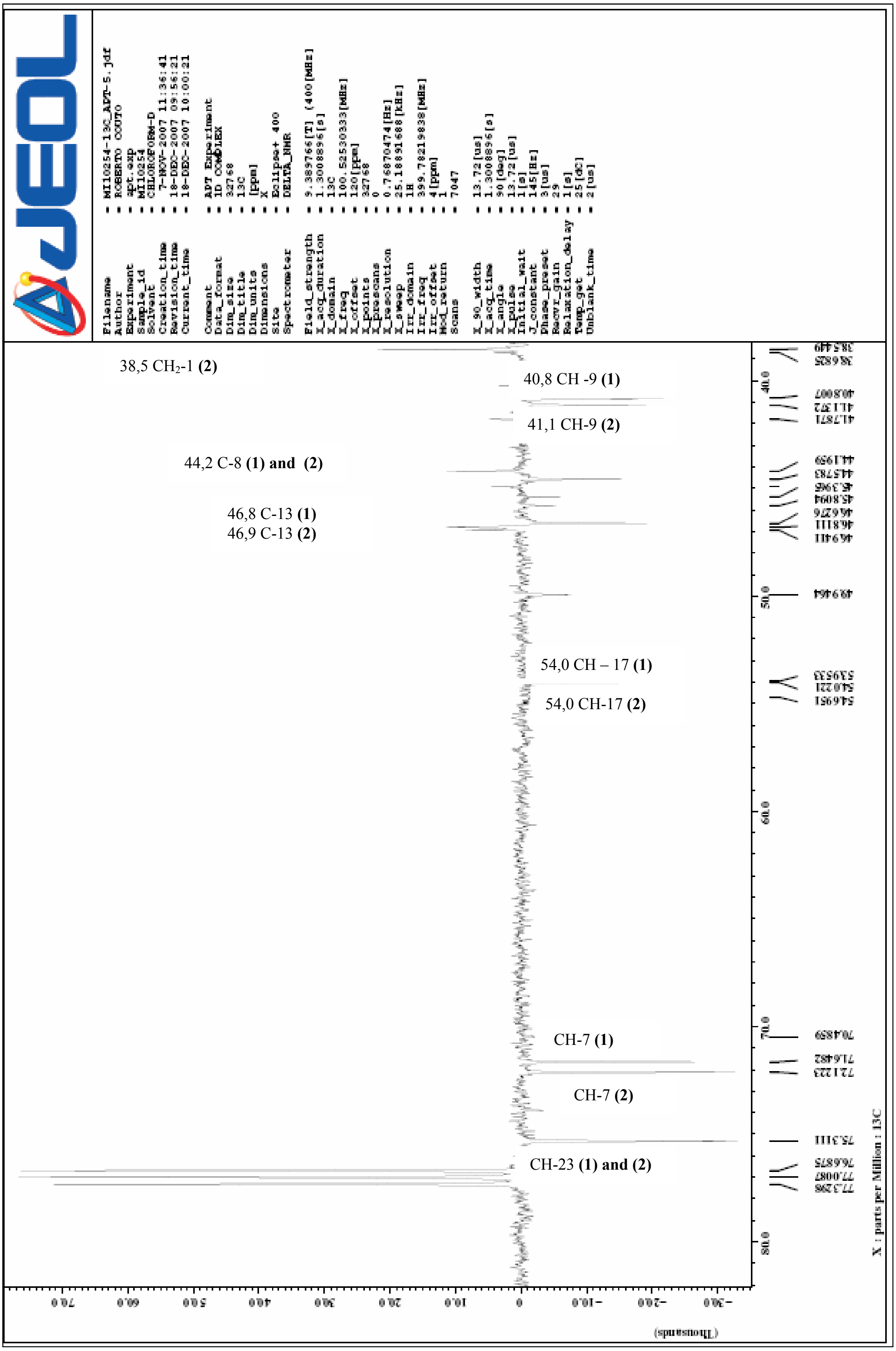

Figure S7. ^{13}C NMR-APT spectrum of protolimonoids **1 and 2** (100 MHz, $CDCl_3$).

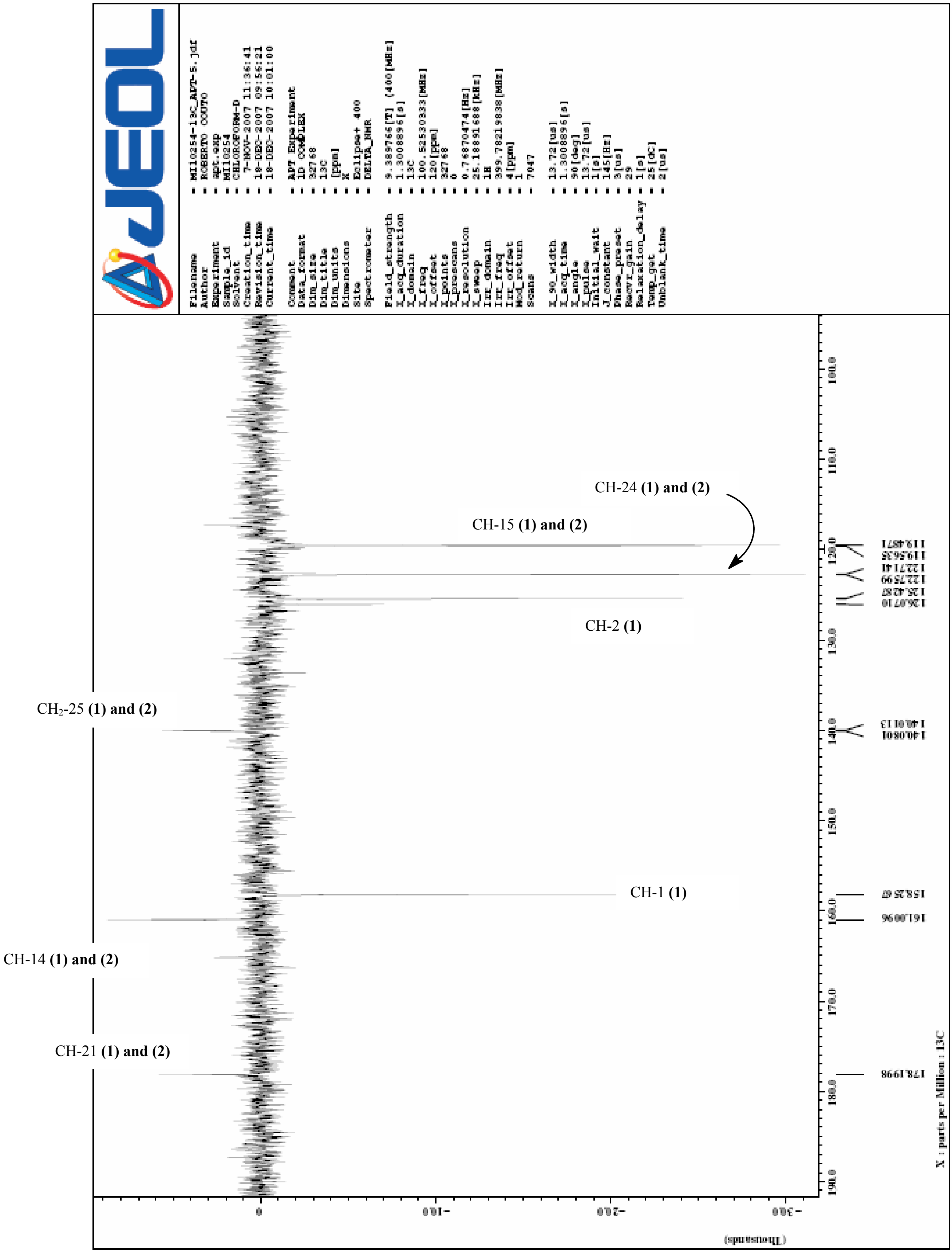

Figure S8. HMBC spectrum of protolimonoids **1** and **2** (400 MHz, $CDCl_3$).

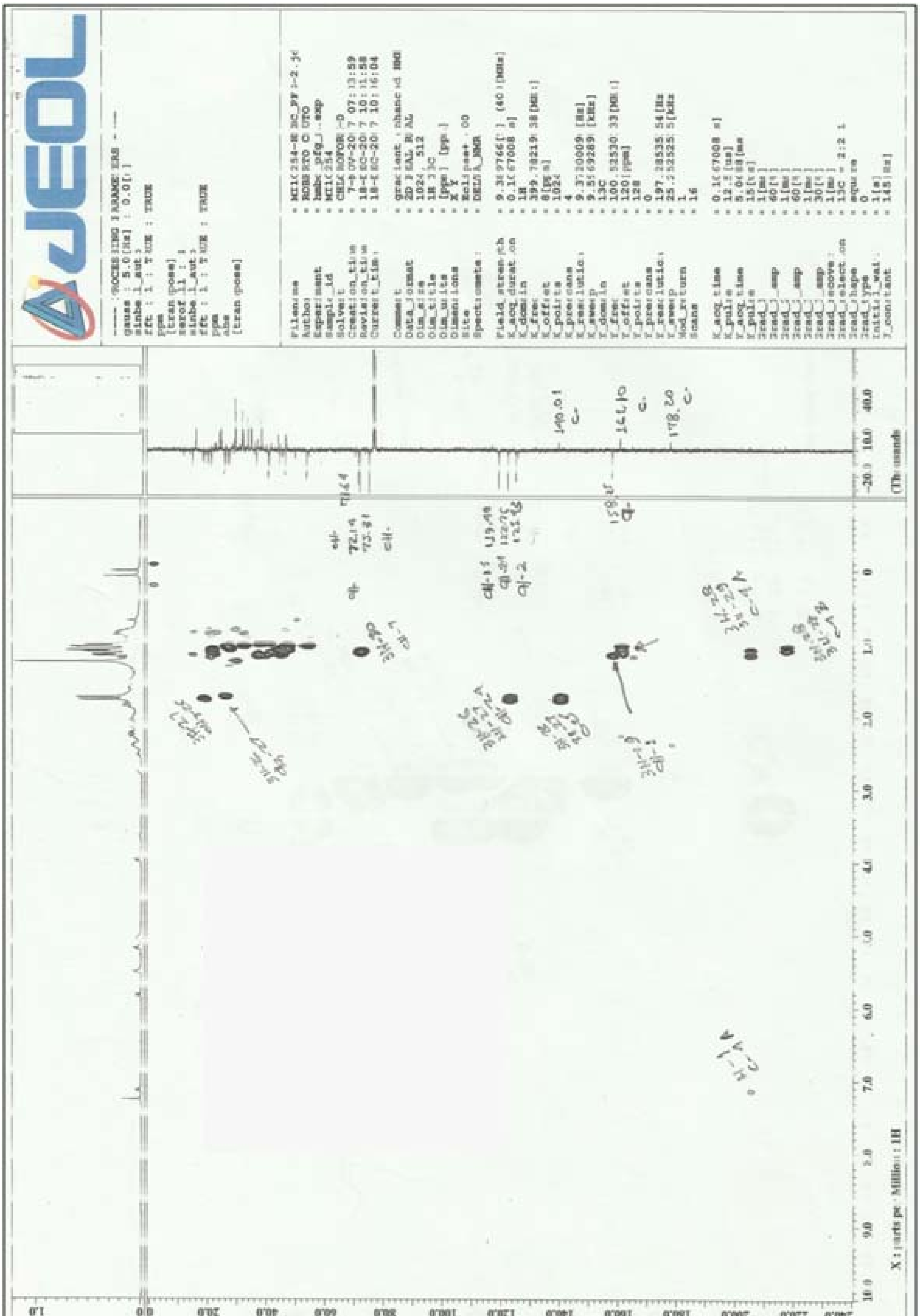

Figure S9. HMBC spectrum of protolimonoids **1** and **2** (400 MHz, $CDCl_3$).

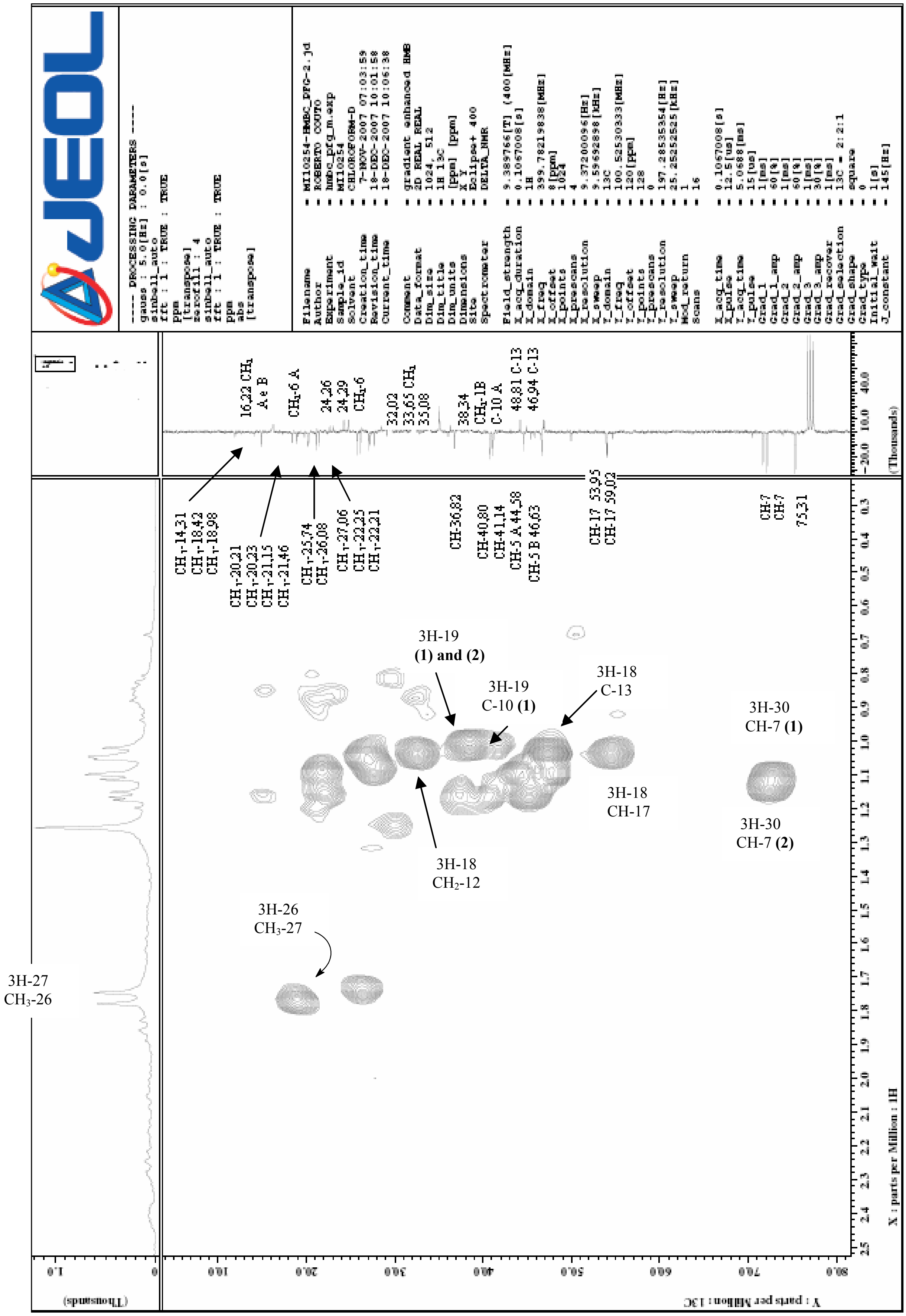

Figure S10. HMBC spectrum of protolimonoids **1** and **2** (400 MHz, $CDCl_3$).

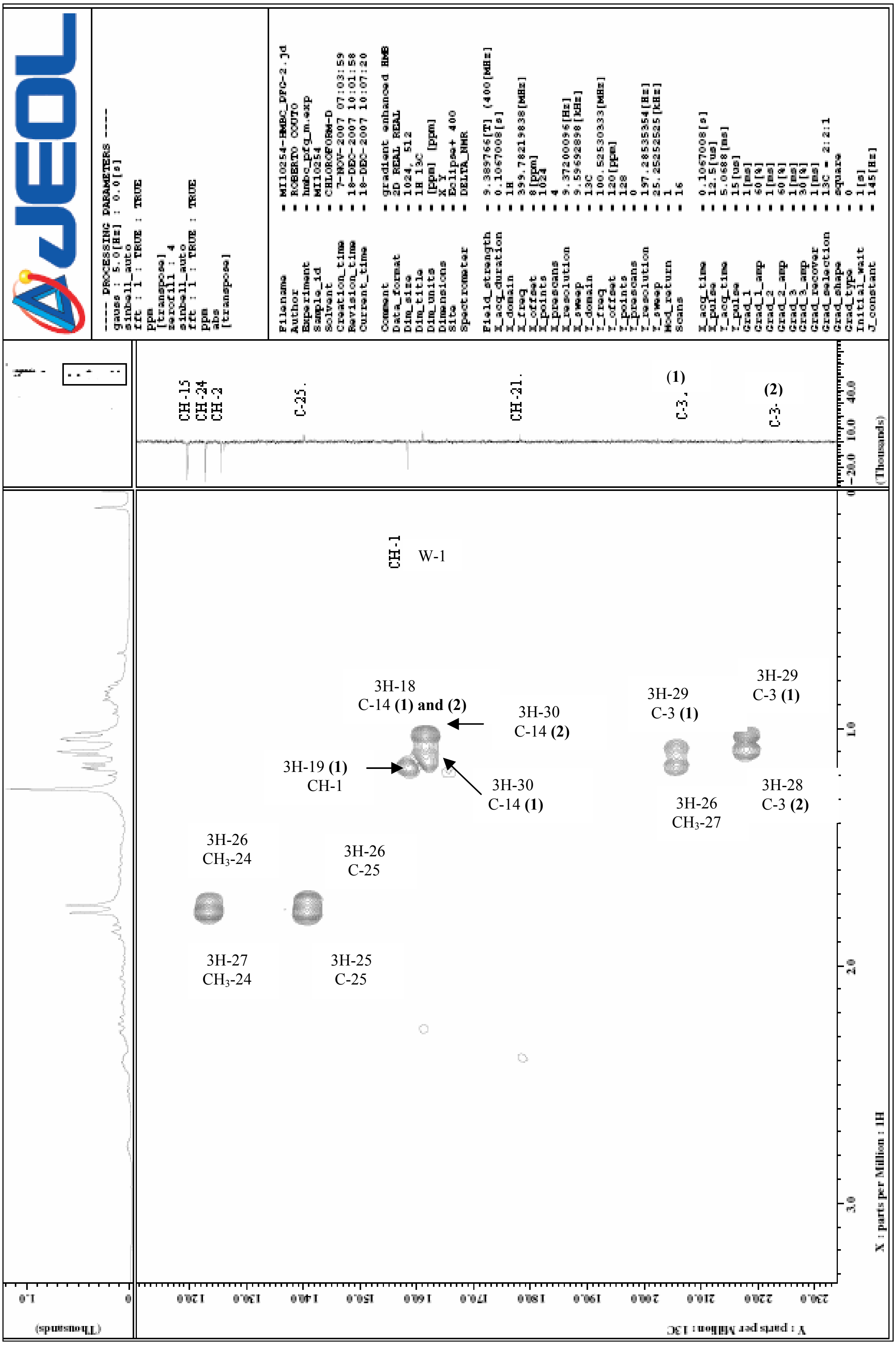

Figure S11. HMQC spectrum of protolimonoids **1** and **2** (400 MHz, $CDCl_3$).

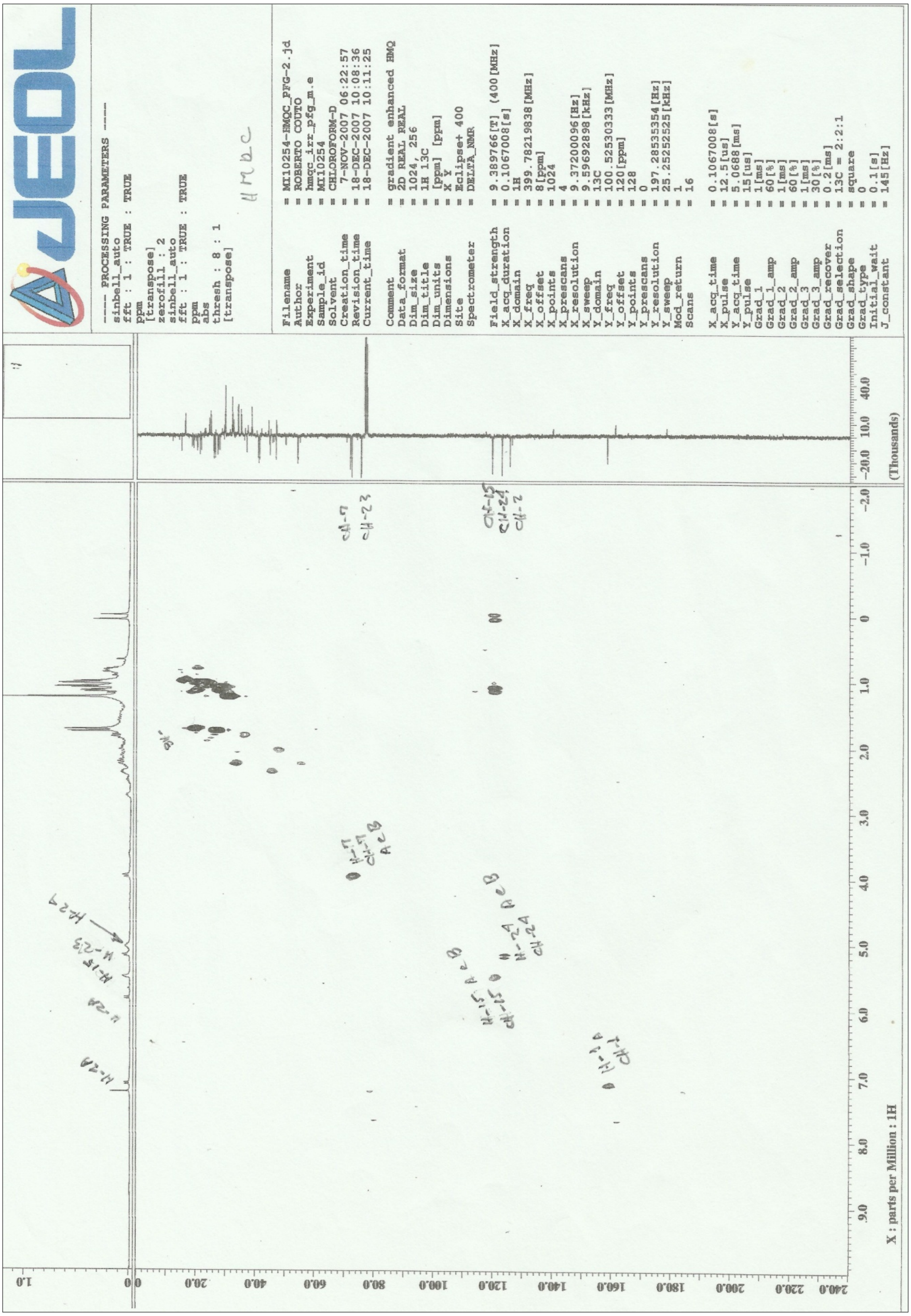

Figure S12. HMQC spectrum of protolimonoids **1** and **2** (400 MHz, $CDCl_3$).

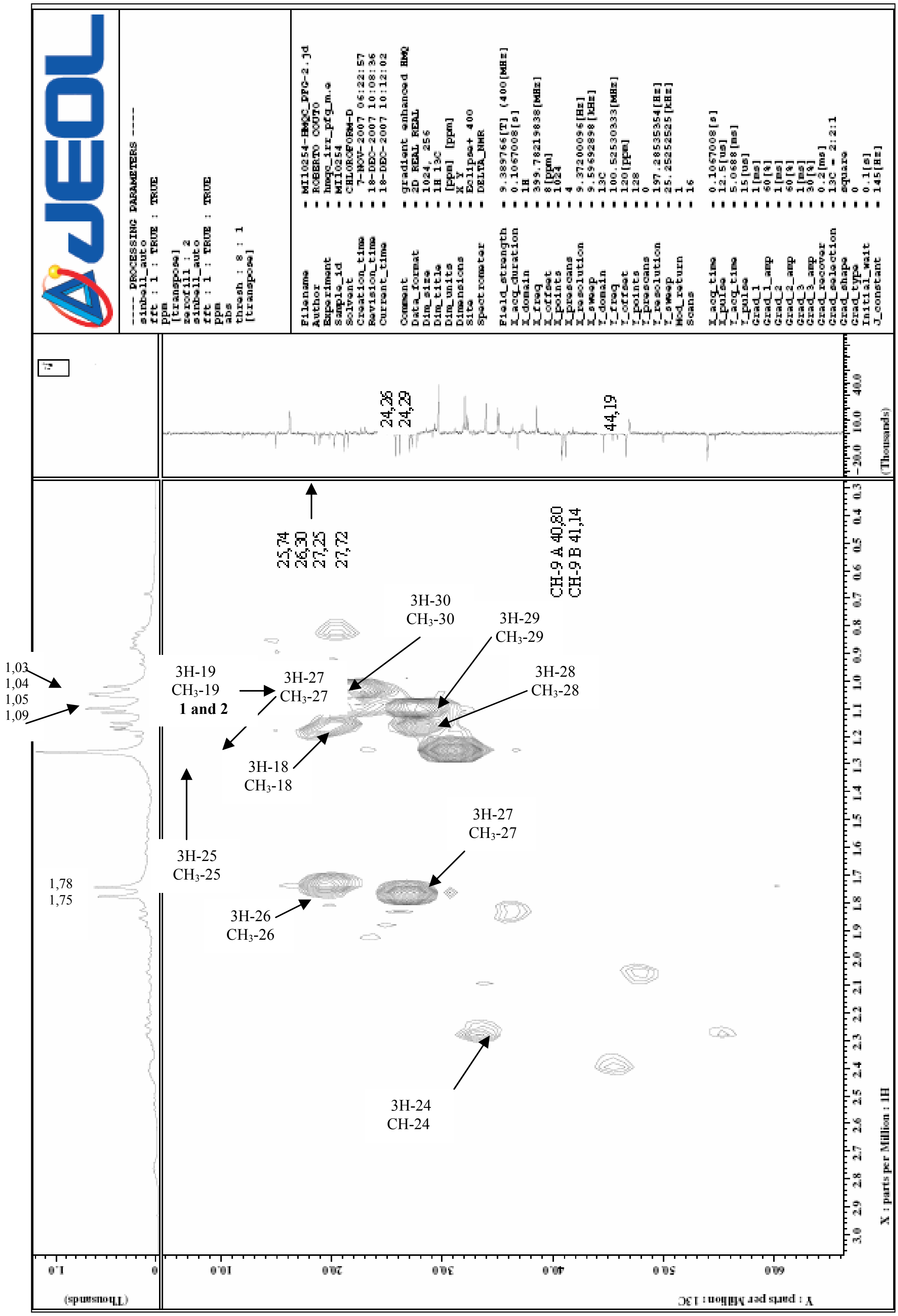

Figure S13. HR-ESI/MS spectrum of protolimonoid **1**.

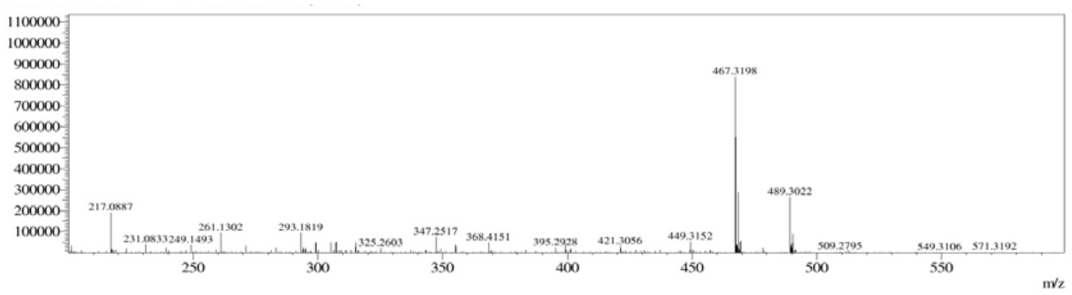

Figure S14. HR-ESI/MS spectrum of protolimonoid **2**.

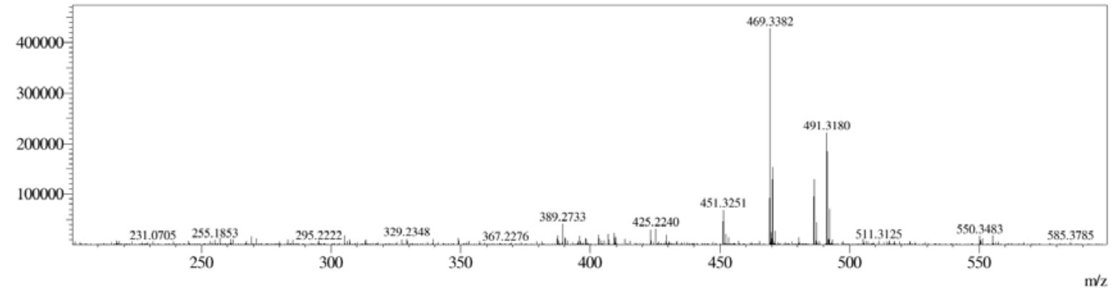

Figure S15. Proposed fragmentation mechanisms to justify the principal peaks observed in the HRESIMS (positive mode) of the mixture of **1** and **2**.

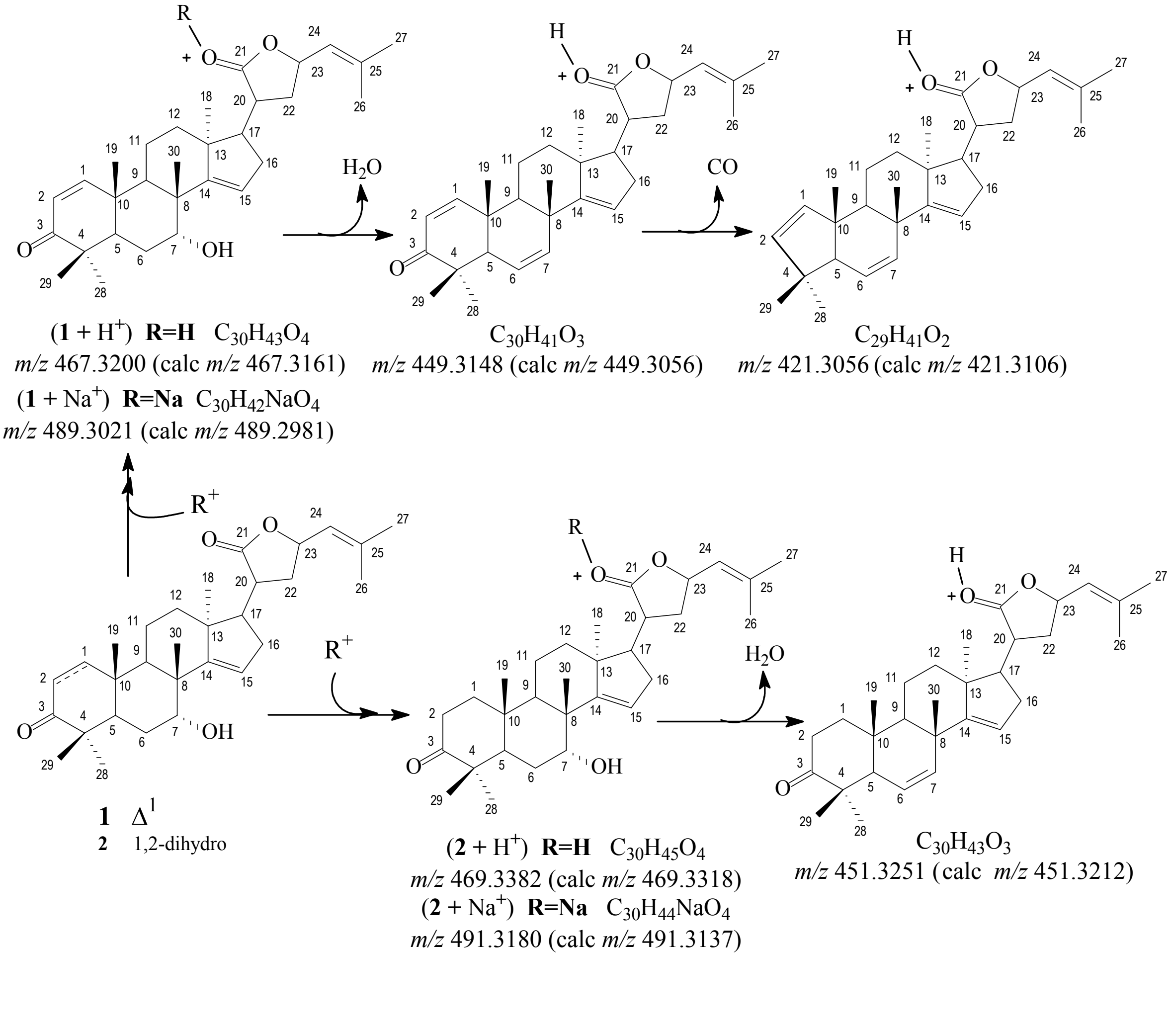

Figure S16. Cellular viability after the treatment with the purified compounds and plant extracts. Leukemia cell lineages U937 and MOLT-4 were incubated for 48 hours at 37ºC with different concentration of samples and the cellular viability was evaluated by MTT assay. A- Mixture of the protolimonoids 1 and 2; B- Protolimonoid 6; C- Methanol extract; D- Hexane extract.

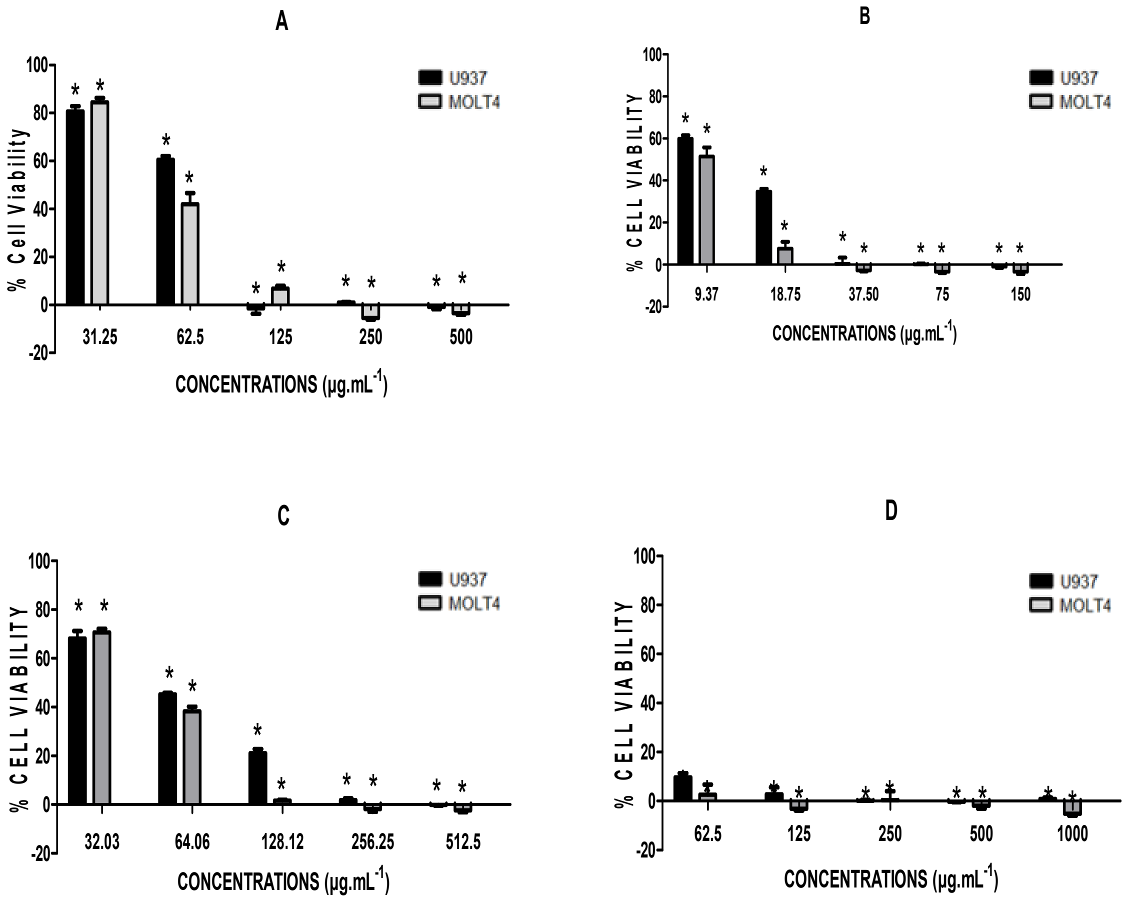

Supplement: Supplementary file 1 [file molecules-18-12180-s001.pdf]
